# Frizzled 8 promotes the cell proliferation and metastasis of renal cell carcinoma

**DOI:** 10.18632/oncotarget.20742

**Published:** 2017-09-08

**Authors:** Qiwei Yang, Ye Wang, Xiuwu Pan, Jianqing Ye, Sishun Gan, Fajun Qu, Lu Chen, Chuanmin Chu, Yi Gao, Xingang Cui

**Affiliations:** ^1^ Department of Urology, The Third Affiliated Hospital of Second Military Medical University (Eastern Hepatobiliary Surgery Hospital), Shanghai 201805, People’s Republic of China; ^2^ Department of Urology, Changzheng Hospital, Second Military Medical University, Shanghai 200003, People’s Republic of China; ^3^ Department of Urology, Changhai Hospital, Second Military Medical University, Shanghai 200082, People’s Republic of China; ^4^ Department of Urology, Ruijin Hospital, Shanghai Jiaotong University, Shanghai 200025, People’s Republic of China

**Keywords:** renal cell carcinoma, Wnt/β-catenin, Frizzled 8, proliferation, metastasis

## Abstract

Recent reports have shown a rapid rise in the incidence of renal cell carcinoma (RCC), and Wnt (Wingless-related integration site) signaling pathway is important in RCC. Frizzled 8 (FZD8) is a member of Frizzled (FZD) receptor family which could activate canonical or non-canonical Wnt/β-catenin pathways. Nevertheless, the role of FZD8 in RCC is poorly investigated. The immunohistochemical analysis showed high expression of FZD8 in RCC tissues compared with peri-tumor tissues. FZD8 knockdown decreased the ability of proliferation and metastasis of RCC cells. Research revealed that the FZD8 regulated the transcription of Cyclin D1, c-Myc, and could promote the epithelial to mesenchymal transition (EMT) by mediating Vimentin and Snail through the Wnt/β-catenin signaling pathway. In addition, the results of our experiment revealed that FZD8 is involved in the regulation of non-canonical Wnt signaling pathway. These data suggested that the expression of FZD8 may play an important role in the proliferation and metastasis of RCC, and serve as a putative promising drug target for human RCC therapy.

## INTRODUCTION

RCC is the common type of malignant tumor in adult urologic neoplasms [1]. The incidence of RCC all over the world has been increasing in recent years [2]. The exact cause and mechanism of development of RCC remains unclear, so its pertinent to study its pathogenesis in greater detail for its eventual prevention and cure. The Wnt signaling pathway has been reported to play crucial role in the development of many tumors [3–6]. Similarly, epithelial to mesenchymal transition (EMT), which is generally accompanied by downregulated E-cadherin and up-regulated β-catenin levels, participate in the tumor progression and tumor metastasis [6–8]. It is reported that von Hippel-lindau (VHL) tumor suppressor gene lost its function in familial and most sporadic clear cell RCC and upregulated β-catenin gives rise to renal carcinoma in mice [9]. Besides, loss of VHL brings about activation of β-catenin by the HGF-driven, which is the degradation complex releasing β-catenin [10].

Wnt/β-catenin signaling is one of the major signaling pathways in cell fate regulation and pathological process. Overexpression of Wnts and FZDs or the expression of a constitutively active FZD regulate various processes which are important for cancer progression, including tumor initiation, growth, and metastasis [11]. The functional versatility of Wnt/β-catenin signaling can be seen as its ability to act in stem cells of embryo and in cancer stem cells of the adult.[12]. Wnt/β-catenin pathway drive metastasis via EMT [13]. and was abnormally activated in a variety of cancers including sporadic clear cell renal cell carcinoma (ccRCC) [14].

Recently, the results of multiple studies have shown that the expression levels of several members of the FZD family, acting in both canonical and non-canonical Wnt signaling pathways, were up-regulated in various cancers. The FZD family includes ten genes in humans and their down regulation inhibits the Wnt signaling pathway leading to the suppression of cell proliferation, migration, invasion, motility and metastasis of tumor cells [15]. For example, FZD2 is reported to be upregulated in the primary Wilms’s tumor, melanoma and squamous cell carcinoma of the lung [16–19]. Also, FZD2 is involved in the regulation of endometrial cancer metastasis by EMT [20] and high-risk neuroblastomas by interfering with β-catenin-dependent and β-catenin-independent signaling pathways [21]. FZD5 was reported to be up regulated in RCC and advanced prostate cancer tissues compared with the normal kidney and benign prostatic hyperplasia tissues [22, 23]. FZD6 was overexpressed in colorectal cancer [24], and it was down-regulated in gastric cancer [25] and leukemia cell lines [26]. Many investigations have found that FZD7 could facilitate invasiveness in Stem-A subtype of ovarian tumor [27], and intensify resistance to targeted BRAF inhibitors in melanoma cells [28]. In addition, knockdown of FZD7 expression in cervical cancer cells also lead to attenuated metastasis and invasion abilities [29], and FZD7 triggered by Wnt3α promotes renal cells proliferation and tumorigenesis [30]. FZD9 was up-regulated in glioblastoma and astrocytoma [31]. MET increased the expression of FZD8 via the ERK/c-Fos cascade in the cancer stem-like cells (CSC) of head and neck squamous carcinomas (HNSCC), and Wnt pathway receptor FZD8 was necessary for interplay between MET and Wnt/β-catenin signaling. The down-regulation of Wnt/β-catenin signaling will bring about growth inhibition and metastasis decrease in the CSC of HNSCC, and the MET/FZD8 signaling axis will become precise therapeutic target of CSC of HNSCC [32]. FZD8 regulated Wnt signaling play a crucial role in promoting resistance to chemotherapy in the triple-negative breast cancer (TNBC) [33]. FZD8 worked as a novel biomarker in the transformation from intestinal metaplasia to gastric cancer [34]. MicroRNA-100 accomplish the repression of migration and invasion of breast cancer cells via stifling Wnt/β-catenin signaling pathway as a result of targeting FZD8 [35]. The lncRNA AK126698 decreases the proliferation and migration of non-small cell lung cancer (NSCLC) cells through targeting FZD8 to inhibit the Wnt/β-catenin signaling pathway [36]. Compared with normal tissue samples, the mRNA levels of FZD5 and FZD8 was up regulated in the RCC tissues from patients [22]. Although members of the FZD family have been widely studied regarding their roles and underlying molecular mechanisms in the development of RCC, as an important receptor of Wnts, the role of FZD8 in RCC has not been characterized. In the present study, we examined FZD8 protein expression in clinical human RCC tissues and peri-tumor tissues, as well as the function of FZD8 in RCC cell lines, which will further explain the tumorigenicity of RCC and provide potential therapeutic target for RCC.

## RESULTS

### FZD8 expression is increased in RCC tissues compared with peri-tumor tissues

**Figure 1:**
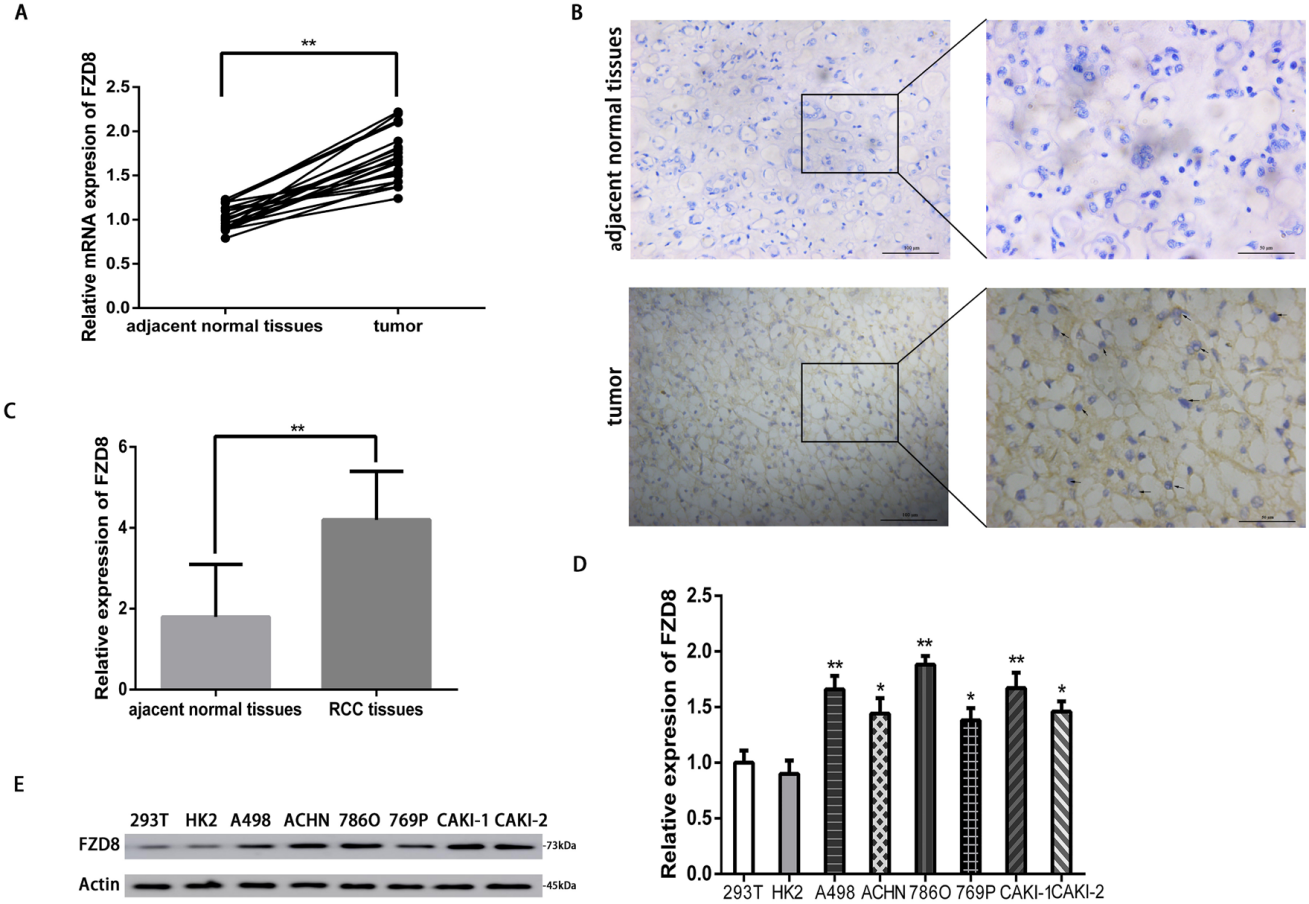
FZD8 is upregulated in RCC tissues **(A)** Real-time PCR analysis of the FZD8 relative expression levels in RCC tissues and adjacent normal tissues from 20 RCC patients. Two-tailed non-parametric Wilcoxon test was used to estimate the significant difference of FZD8 levels. The FZD8 mRNA expression level of RCC tissues was much higher than the adjacent normal tissues (**P<0.01). **(B)** Immunohistochemistry analysis of FZD8 in RCC tissues and adjacent normal tissues. The FZD8 protein expression of RCC tissues was higher than adjacent normal tissues. **(C)** Statistical analysis of immunohistochemical comparison of FZD8 expression in the human RCC tissues versus peritumoral normal tissues (**P<0.01). **(D)** Real-time PCR analysis of the relative expression levels of FZD8 in six human RCC cell lines (A498, ACHN, 786O, 769P, CAKI-1, CAKI-2) versus human fetus normal kidney epithelial cells (293T) and adult kidney cortex/proximal tubule epithelial cells (HK2). The error bars represent the mean±standard deviation (SD) of 3 independent experiments. Specifically, FZD8 expression was highest in 786O cells (*P*<0.01), whereas 769P cells expressed relatively low levels of FZD8 (*P*<0.05). Actin was used as a loading control for all samples. *P < 0.05, **P < 0.01, and ***P<0.001. **(E)** Western blot analysis of protein levels of FZD8 in these cell lines. The WB results were conformed to the consequence of qPCR.

A total of 20 samples were collected from RCC patients receiving radical nephrectomy or partial nephrectomy. The clinical and pathological information of the RCC tissues and peri-tumoral tissues were exhibited as the Supplementary Table 1. To determine the expression levels of FZD8 in RCC, we performed a quantitative reverse transcription-polymerase chain reaction (qRT-PCR) in 20 pairs of RCC samples and adjacent non-tumor samples. The relative mRNA expression levels of FZD8 were found to be increased significantly in the RCC samples (*P<0.01*), compared with those in the matching adjacent non-tumor tissues (Figure 1A). To further investigate the increased expression of FZD8 in RCC tissues, we performed immunohistochemical staining for FZD8, and detected that FZD8 protein expression was highly increased in RCC samples, compared with matching non-tumor samples (Figure 1B). The statistical analysis of immunohistochemical results were showed on the Figure 1C. Furthermore, the expression of FZD8 was observed in diverse RCC cell lines. Compared to normal renal cell lines, the mRNA levels of FZD8 was increased in RCC cell lines (Figure 1D). The WB analysis of the FZD8 expression in A498, ACHN, 786O, 769P, CAKI-1 and CAKI-2 cells were higher than 293T and HK2 cells as presented in the Figure 1E, which indicated the protein levels of FZD8 was confirmed to the qRT-PCR result.

### FZD8 promotes RCC cell proliferation through the canonical and non-canonical Wnt signaling pathway

**Figure 2:**
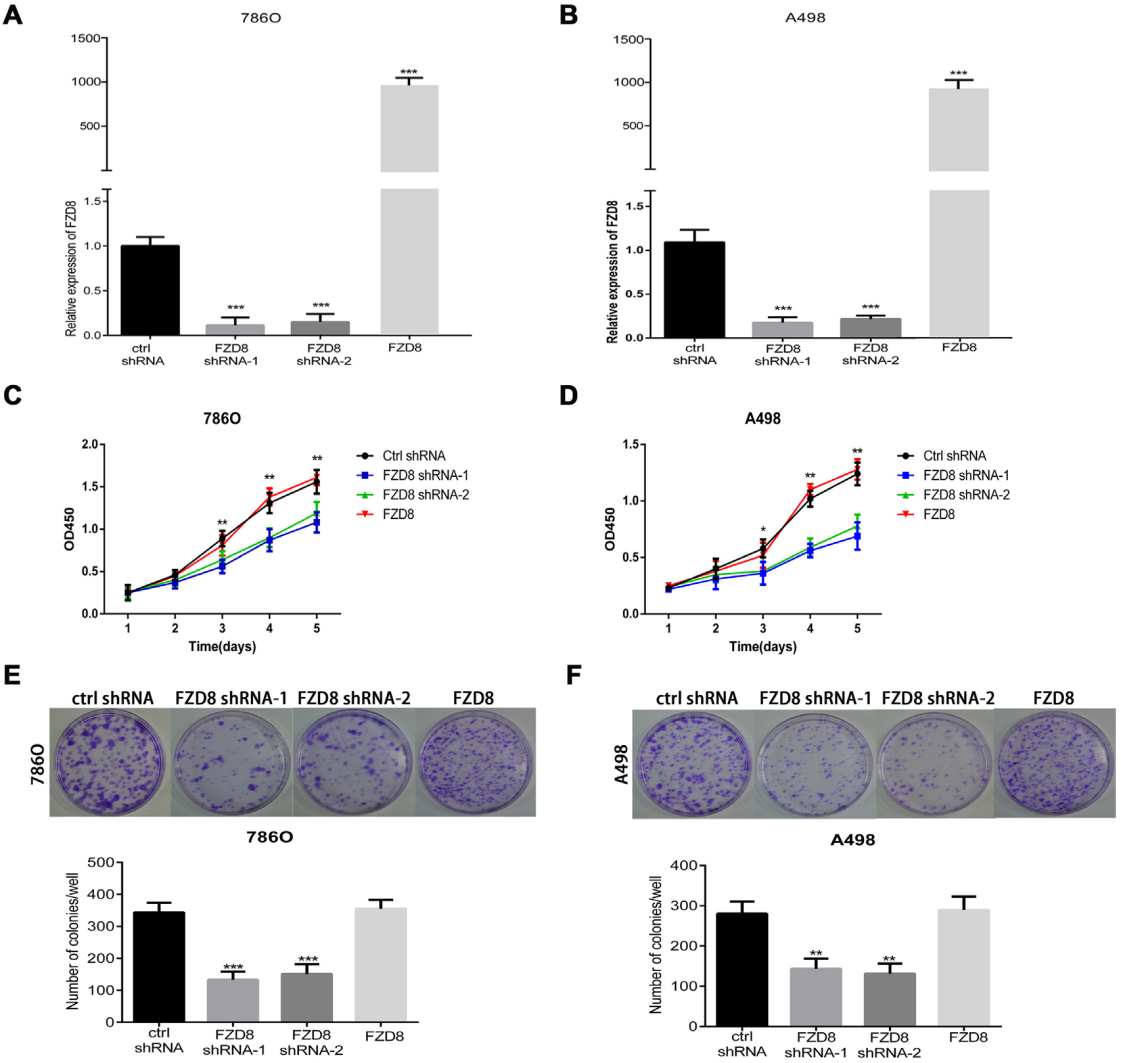
FZD8 facilitates RCC cells proliferation *in vitro* (**A** and **B**) qRT-PCR analysis of FZD8 in control, FZD8 knockdown and FZD8 overexpression (786O and A498) cells. **(C)** CCK8 assay of FZD8 knockdown, overexpression and control 786O cells at the appointed time points. The infected cells were seeded to 96-wells plates. After incubation for several days, the cell growth was measured by CCK-8 assay. In FZD8 downregulated 786O the cell growth was suppressed in a time-dependence manner. After 24h and 48h incubation, no difference in cell viability between two groups was observed (*P*>0.05). Nevertheless, the markedly difference between the two groups was observed after 72h, 96h and 120h (*P*<0.001, respectively). The FZD8 overexpression and control group showed no significant difference in cell viability at different time points. **(D)** The growth of FZD8 downregulated A498 cells were slower than the control cells. Similarly, it was no difference in cell viability between two groups in cell viability after 24h and 48h cultivation (*P*>0.05). Nonetheless, it was significant difference in cell viability between the two groups after 72h (*P*<0.05), 96h and 120h cultivation (*P*<0.01, respectively). Similarly, the overexpression of FZD8 also have minimal effect on the cell viability during the entire cell growth period compared with control group. (**E** and **F**) Plate colony formation assay for 786O and A498 cells stably transfected with control or FZD8 shRNAs in 6 cm dish for 2 weeks. Experiments were performed in triplicate. Results are means±S.D. (error bars). The images of colony formation assay were taken under a light microscope (20×). Representative images (upper) and average number of colonies (lower) were shown. Plate colony formation assay demonstrated that downregulation of FZD8 expression in those stable lines led to significant proliferative inhibition when compared to the controls (786O: *P*<0.001, A498: *P*<0.01), and the number of colonies between the group of FZD8 overexpression and control exhibited no significant difference.

To study the biological function of FZD8 in RCC cells further, FZD8 targeted lenti virus-based short hairpin RNAs (shRNAs) were used to silence FZD8 mRNA expression. In order to confirm the target effects produced by shRNA, we used qRT-PCR that both stably transfected FZD8 shRNA and knockdown the FZD8 expression in 786O and A498 cell lines expressing the gene, whereas the scramble control shRNA had no effect (Figure 2A and 2B). According to the interference efficiency of the two FZD8 shRNAs, we selected FZD8 shRNA-1 and FZD8 shRNA-2 in all experiments. The proliferative function of FZD8 could be characterized by the CCK8 assay and plate colony formation assay. As shown in Figure 2C and 2D, the knockdown of FZD8 resulted in decreased proliferation of RCC cells. The number and size of single colony was also reduced in FZD8 knockdown (Figure 2E and 2F).

**Figure 3:**
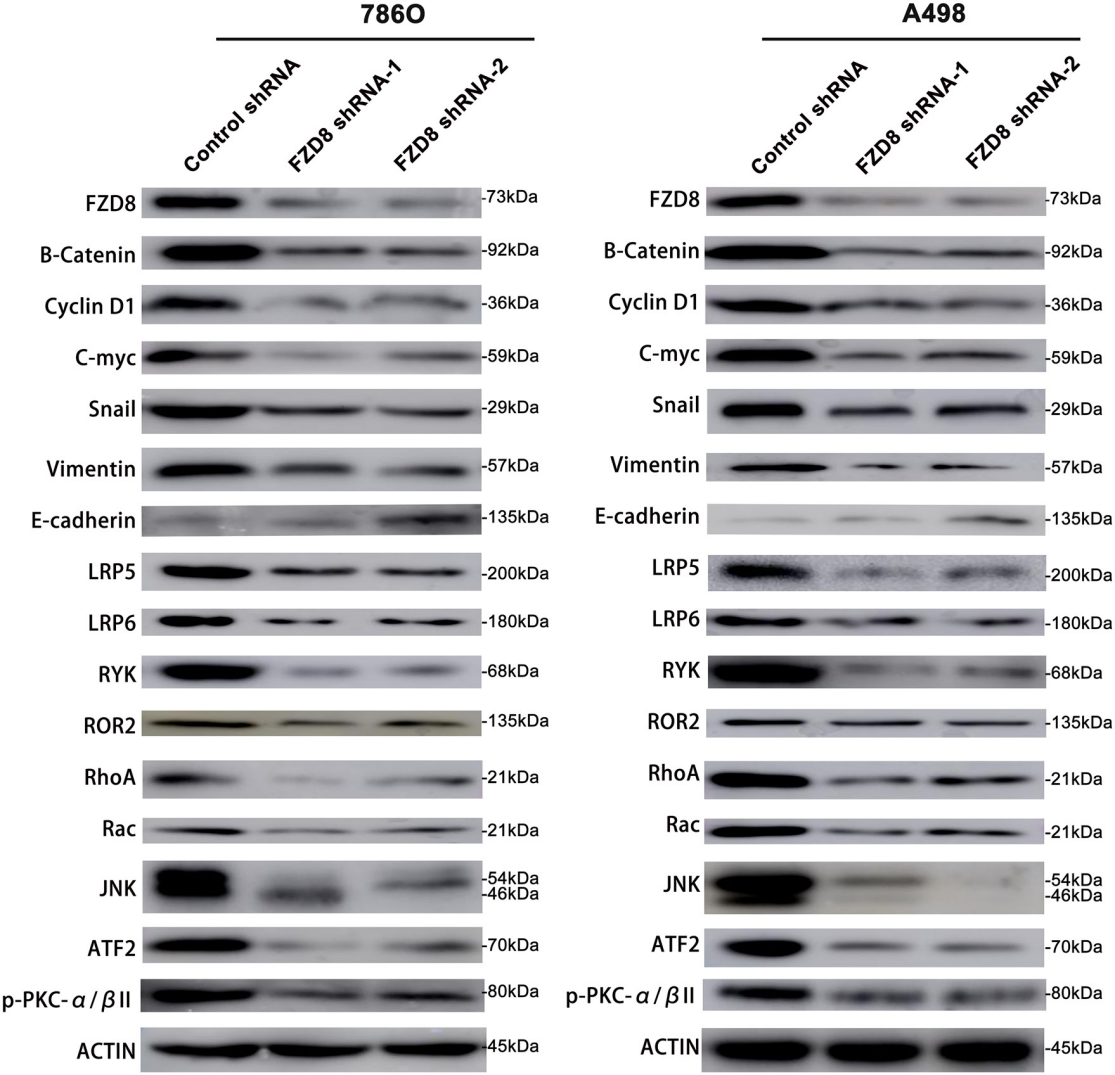
Western blotting analysis of FZD8, β-catenin, Cyclin D1, C-myc, Snail, Vimentin, E-cadherin, LRP5, LRP6, RYK, ROR2, RhoA, Rac, JNK, ATF2, p-PKC-α/βII and Actin in FZD8 knockdown and control groups Western blot analysis showing that FZD8 inhibition by shRNAs decreased FZD8, β-catenin, Cyclin D1, C-myc, Snail, Vimentin, LRP5, LRP6, RYK, ROR2, Rho A, Rac, JNK, ATF2, and p-PKC-α/βII protein levels, and increased the E-cadherin protein level in 786O and A498 cells as compared with control shRNA treated cells.

Then, we examined the effects of FZD8 expression in knocking-down, the canonical Wnt signaling pathway in RCC cells. FZD8 shRNA-1 and FZD8 shRNA-2 substantially decreased FZD8 protein levels compared to control group (Figure 3). The cytosolic level of β-catenin protein, an important signature of the canonical Wnt activation, could reveal the effect of FZD8 on Wnt/β-catenin signal transduction. Western blot analysis, which revealed the decreased level of cytosolic β-catenin protein after the FZD8 shRNA-1 and FZD8 shRNA-2 were transfected as compared with control shRNA-expressing control cells (Figure 3), indicated that FZD8 function as a positive regulator of the canonical Wnt/β-catenin signaling pathway in RCC cells. We then evaluated the target genes of the Wnt pathway, such as Cyclin D1 and C-myc, which were downstream components of the Wnt pathways. We found that the levels of both Cyclin D1 and C-myc were down-regulated after the treatment of FZD8 shRNAs (Figure 3). Taken together, these results suggest that FZD8 may be functionally significant for aberrant activation of the canonical Wnt signaling pathway in human RCC cells.

In the study of FZD8 co-receptors, we were focused on the lipoprotein (LDL) receptor-related protein-5 (LRP5), LRP6, RYK, and receptor tyrosine kinase-like orphan receptor (ROR) 2. In the previous studies reports, the low-density LRP5 regulates the osteoblastic metastases and the tumor caused new bone formation in prostate cancer [37]. LRP6 facilitates oncogenesis by mediating Wnt/β-catenin subcellular localization in fibrosarcoma [38]. RYK is involved with many signaling pathways in the neoplasia of ovarian tumor [39]. The expression of ROR2 was increased in the chemoresistant ovarian cancer and mediate cell migration and invasion via EMT [40]. In our experiments, the expression of LRP5, LRP6, RYK and ROR2 were down regulated after the konckdown of FZD8 levels in 786O and A498 cells as shown in Figure 3, which indicate that FZD8 facilitate the RCC proliferation and metastasis is also dependant on the coreceptor of frizzled family members.

We also detected the non-canonical Wnt signaling pathway variation after the knockdown of FZD8, and previous investigation have demonstrated two non-canonical pathways, which include the Wnt polarity (PCP) pathway and the Wnt/Ca^2+^ pathway [41]. The PCP pathway is depending on Rho A and JNK activation [42], and in the Wnt/Ca^2+^ pathway, intracellular stores could liberate the calcium, which activates the protein kinase C (PKC) causing the enhanced migration and invasion of oral squamous cell carcinoma cells [43]. We test the variation of important members in the non-canonical signaling pathway following the knockdown of FZD8, the levels of RhoA, Rac, JNK and ATF2 were downregulated in the PCP pathway and phosphorylate-PKC-α/β II expression were reduced in the Wnt/Ca^2+^ pathway as shown in Figure 3.

### FZD8 facilitates RCC cell migration and invasion

**Figure 4:**
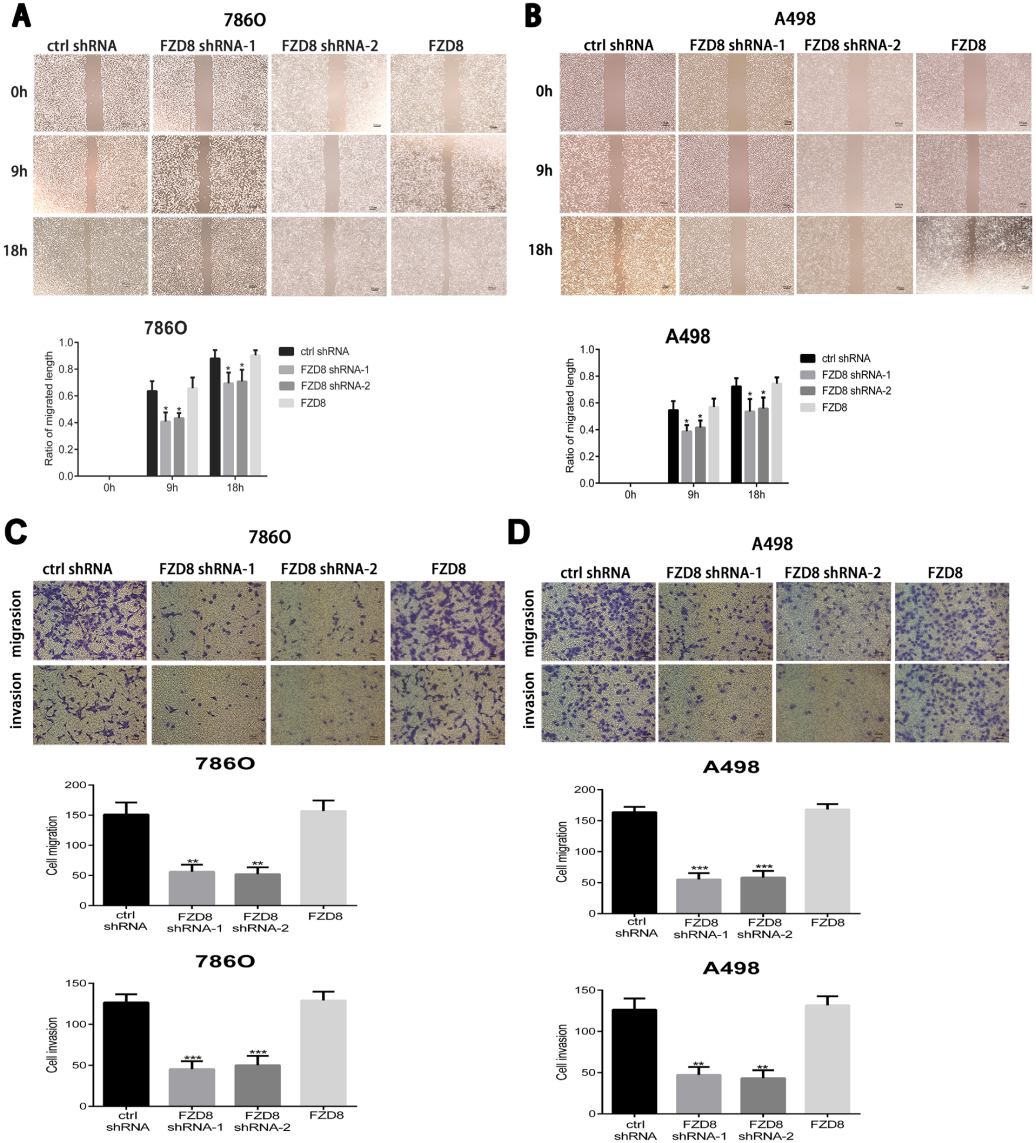
FZD8 promotes RCC cells migration and invasion *in vitro* (**A** and **B**) Up images: representative images of the wound-healing assay of FZD8 control, knockdown and overexpression (786O and A498) cells photographed at 0, 9 and 18 h after scraping. Scale bar = 100μm. Down images: the relative migration rate was computed by separating the change in the distance between the scrape edges by the initial distance. The wound-healing rate was counted at 0, 9, 18 hours after scratching. The knocking-down of FZD8 obviously repressed the migration of 786O and A498 cells compared with the control group (vs. control shRNA groups; *P* < 0.05, respectively), and the migration distance between the control and FZD8 over-expression groups displayed little significant difference. **(C)** Matrigel-coated transwell assays were performed to estimate the invasion (lower), and non-coated Transwell assay were performed to estimate migration (upper), following FZD8 knockdown and control 786O cells. The statistical graph exhibits the means±SD of the number of cells from 10 random high power fields (magnification, ×100) counted from three independent experiments. Scale bar = 100 μm. The down-regulation of FZD8 markedly decreased cell migration and invasion (vs. control shRNA; p < 0.01 and 0.001, respectively), and the number of transferred to the bottom cells between control and FZD8 overexpression groups showed no significant difference. **(D)** The migration and invasiveness of A498 cells after transfection with FZD8 shRNAs were also investigated. The knock-down of FZD8 remarkably reduced cell migration and invasion (vs. control shRNA group; *P* < 0.001 and *P* < 0.01, respectively), and the FZD8 overexpression have little effect on the cell migration and invasion.

The metastasis is the main reason for RCC patients’ poor prognosis. To investigate the effects of FZD8 in RCC metastasis *in vitro*, the wound healing assay and transwell assay were both performed to characterize the role of FZD8 in RCC cell metastasis. Wound-healing assay showed that FZD8 knockdown inhibited cell migration in RCC cells (Figure 4A and 4B). Transwell assay demonstrated that FZD8 knockdown significantly suppressed migration and invasion of renal cells (Figure 4C and 4D).

To further validate the role of FZD8 as described above, we examined the levels of EMT markers. As shown in Figure 3, we observed FZD8 knockdown increased epithelial markers (E-cadherin) expression and decreased levels of mesenchymal markers (Snail and Vimentin).

### Up-regulation of FZD8 has minimal effect on RCC cell proliferation and metastasis

To further validate that FZD8 promotes RCC proliferation, as described above, we upregulated the levels of FZD8 in 786O and A498 cells (Figure 2A and 2B). As shown in Figure 2C and 2D, we did not detect the increase or decrease of proliferation in 786O and A498 cells following the overexpression of FZD8. The plate colony formation assay also confirmed the results of CCK8 assays as shown in Figure 2E and 2F.

In order to test the effects of FZD8 upregulation in the metastasis of RCC cells, we also performed the wound healing experiments and transwell assays after the upregulation of FZD8 in 786O and A498 cells. As shown in the Figure 4A and 4B, the overexpression of FZD8 did not improve or inhibit the migration of RCC cells. The results of transwell migration assay also confirmed the outcomes of wound healing expriments as shown in Figure 4C and 4D. Matrigel invasion chamber assay detected that FZD8 overexpression did not enhance or attenuate invasion as displayed in Figure 4C and 4D.

### FZD8 knocking-down suppressed growth of renal cancer *in vivo*

**Figure 5:**
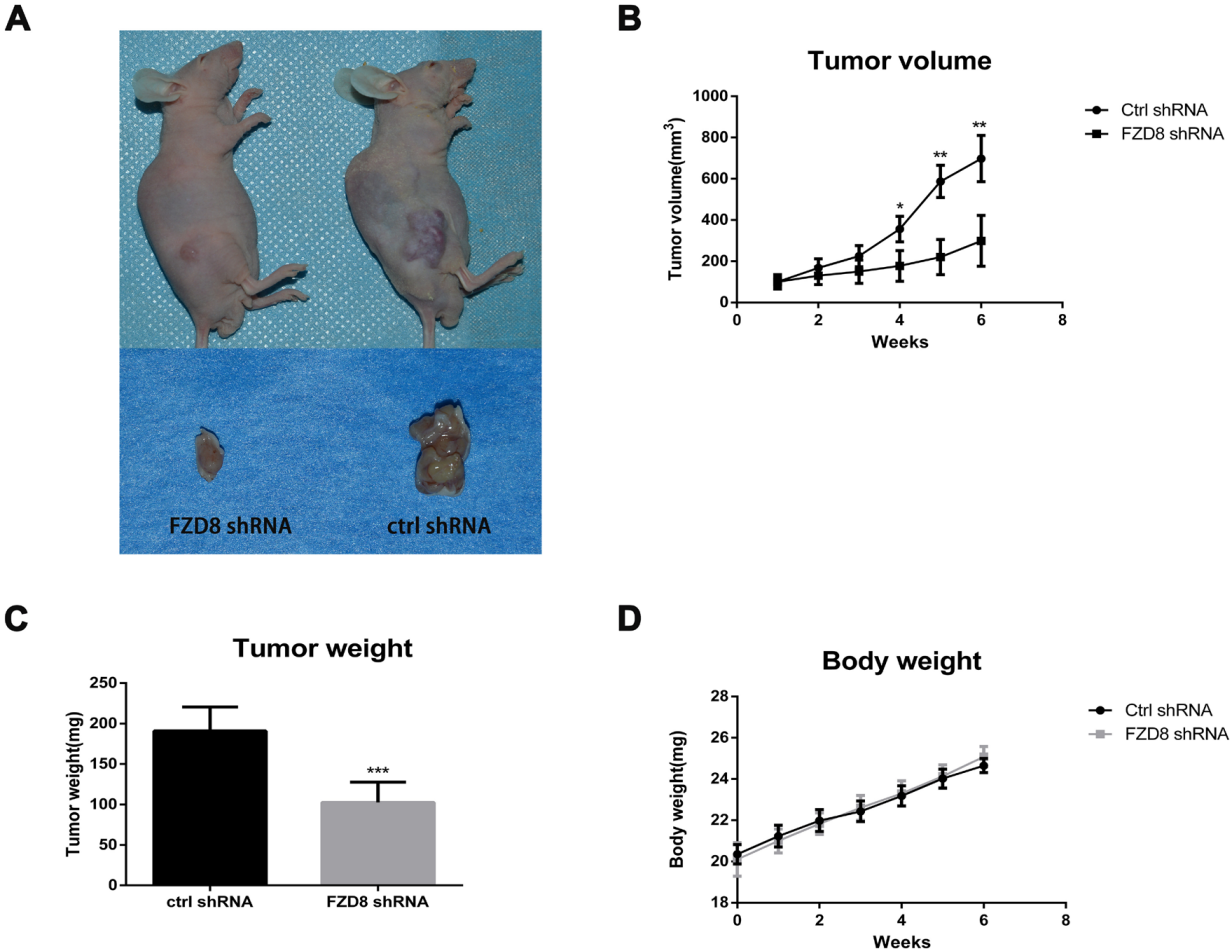
FZD8 knockdown represses tumor progression *in vivo* **(A)** Two representative nude mice demonstrating the morphology of the tumors derived from FZD8 knockdown (left) and control 786O cells (right). Six weeks after inoculation, Tumors in both groups were euthanized to dissect and photograph (lower). Xenograft formation was notably decreased in mice bearing FZD8 knockdown cells compared with mice bearing control shRNA vector cells. **(B)** Tumor size was monitored and measured every 3 days after subcutaneous implantation of 786O cells stably transfected with FZD8 shRNA-1 or control shRNAs. Tumor volume was calculated by using the equation width^2^×length/2. The growth curve of the FZD8 knockdown versus the control cells was performed according to the tumor volumes of indicated times. The significant decrease in tumor size of the FZD8 shRNA tumors (N=5) compared to those of the control shRNA tumors (N=5) (**P<0.01). **(C)** The average weight of FZD8 knockdown group mice versus the control 786O tumors. Tumor weight was weighed up at the accomplishment of the experiment. Tumor weight of FZD8 shRNA treated group was markedly less than that of the control shRNA treated group (***P<0.001). **(D)** Body weight was checked every 3 days after the FZD8 shRNA-1 786O cells and control shRNAs 786O cells were injected subcutaneously. The body weight curve was produced according to the periodical measurements when the tumors could perceptible.

Finally, we established xenograft mouse models with 786O cells stably transfected with control or FZD8 shRNA-1. The stable lines were implanted into female BALB/c nude mice. Then tumor formation was monitored and tumor mass was measured every 3 days. As shown in Figure 5A, 5B and Supplementary Table 2, we observed a significant reduction in tumor size and mass of the FZD8 knocking-down group compared to those of the control group (N=5). After 8 weeks of tumor growth, mice were sacrificed and the tumors were collected for weight measurement. We found that tumor weight of FZD8 knocking-down treated groups was significantly less than that of the control treated group (*P*<0.001) (Figure 5C and Supplementary Table 2). The body weight of two groups of mice have not detected significant difference as shown in Figure 5D and Supplementary Table 2.

## DISCUSSION

RCC metastasis is the primary reason of death in RCC patients, but the underlying mechanisms remain uncertain. So, it is urgent to probe the substantial cause of the metastatic initiation and development, which prevents the metastasis in the early stage of RCC progression. In addition to the well-known signaling pathways such as VHL, VEGFR, and mTOR, Wnt/β-catenin has become a potent pathway for RCC pathogenesis [44]. Glycoprotein Wnts are secreted proteins, related to carcinogenesis and development of fetus [45] via attachment to the Frizzled (FZD) receptor family [46]. Upon attachment of the Wnt proteins to the FZD family protein receptors on the cell membrane surface, the canonical and non-canonical Wnt signaling pathways are activated [46]. In the canonical signaling pathway, the connection of Wnts with FZD co-receptor and alternatively low-density lipoprotein receptor-relevant proteins, such as LRP5 or LRP6, reduces the degradation of β-catenin, inducing the nuclear transition and steadiness of β-catenin [47]. In the breast cancer, LRP6 is upregulated in the patients’ tissues and tumor cell lines, promoting tumor migration and invasion via Wnt/β-catenin signaling pathway [48]. Co-receptor LRP5 and LRP6 could interplay between themselves and attach to FZD and Wnt ligands, prohibiting FZD mediated canonical and non-canonical pathways and further regulating tumor metastasis [49]. RYK facilitate the retaining of stemness and tumorigenic traits of glioblastoma multiforme and glioma cells, controlling its role via β-catenin signaling. Moreover, RYK is upregulated in the glioblastoma stem cells, which is essential for the stimulation of the pluripotency-relevant transcription-factor circuitry, and supporting neurosphere formation, resulting in an in-distinguished condition [50]. High expression of ROR2 is upregulated in non-small cell lung cancer (NSCLC) [51] and cervical cancer [52], and is also related to advanced TNM stage indicating poor prognosis. Downregulating the expression of ROR2 significantly suppressed cancer cell migration and invasion, and overexpression of ROR2 in parental cell line promoted the cell ability of invasion [53]. The canonical signaling pathway contain different functions, such as cell development direction, cell vitality, cell proliferation [54], carcinogenesis and metastasis [55]. Nevertheless, the non-canonical signaling pathway of Wnts is not depend on β-catenin. Previous studies have illustrated two non-canonical pathways, which contain the Wnt/Ca^2+^ pathway and the Wnt polarity (PCP) pathway [41, 42]. In the Wnt/Ca^2+^ pathway, the protein kinase C (PKC) were activated by the calcium, which released from cellular storage, leading to the facilitated migration and invasion of oral squamous cell carcinoma cells [43]. The PCP pathway is relaying on RhoA and JNK activation [42], and more and more study have reported that Wnt signaling also participate in altering tumor cell metabolism to control cancer bioenergetics [56].

The RhoA protein prohibits apoptosis and promotes proliferation of incubatory SPCA1 lung cancer cells [57], and downregulation of RhoA suppresses the neoplastic overgrowth in Asian gastric cancer [58]. Knockdown of Rac inhibits proliferation and metastasis of human breast cancer [59]. Investigation suggested that JNK play pro-oncogenic role in the progression of cancer, while others believed that JNK worked as a cancer inhibitory factor [60]. ATF2 facilitates RCC cells proliferation and metastasis [61]. The results of our experiments as displayed in the Figure 3, demonstrated that the inhibition of FZD8 could down regulate the expression of co-receptors of frizzled family and downstream components of canonical and non-canonical signaling pathway, which could prohibit the RCC cells proliferation and metastasis.

Previous studies have demonstrated the upregulated expression of FZD8 in primary lung tumor, and knockdown of FZD8 inhibited the growth of lung cancer and make lung cancer cells sensitive to taxotere therapy [62]. In triple-negative breast cancer (TNBC), the knockdown of FZD8 results in enhanced apoptosis with down-regulated β-catenin and survivin expression, and sensitizes the TNBC cells to chemotherapy, such as cisplatin plus TRAIL [33]. The knockdown of MET/FZD8 pathway in head and neck squamous carcinomas (HNSCC) diminishes the cancer stem-like cells (CSC) population and prohibits the progression and metastasis of HNSCC [32], FZD8 expression was found to be up-regulated in renal cell carcinoma, acute lymphoblastic leukemia and lung cancer, but the function of FZD8 in the development of RCC is poorly investigated [22, 62, 63]. In the present study, we examined the expression of FZD8 mRNA and protein in patients with RCC and found it to be upregulated in tumors, compared with non-tumor tissues. The expression of FZD8 is required for the activation of genes associated with the canonical and non-canonical Wnt signaling pathway.

We also investigated whether FZD8 expression was associated with aberrant Wnt pathway activation and cell proliferation, invasiveness and metastasis in renal cancer. We demonstrated a significant overexpression of FZD8 in primary renal cancer tissue samples when compared to their adjacent normal tissues. This observation suggests that FZD8 makes great contribution in human RCC as it is one of the important receptor for transducing Wnt signaling to activate downstream canonical and non-canonical Wnt cascade. To test the possible functional significance of the FZD8 overexpression in RCC, we used shRNA and stable transfection technologies to knockdown endogenous FZD8 expression in 786O and A498 cell lines. We observed that knocking down FZD8 expression in the two cell lines not only inhibited proliferation *in vitro*, but also suppressed tumor growth *in vivo*. Knocking down FZD8 expression was also accompanied by inhibition of canonical Wnt signaling in these cells as evidenced by a decrease in the levels of cytosolic β-catenin and its downstream target genes such as cyclin D1 and c-Myc. Aberrant activation of non-canonical Wnt signaling pathway has also been shown to directly promote the invasiveness and malignant progression of various types of cancers in similar lines as canonical Wnt signaling pathway. In our study, downregulation of FZD8 levels also led to prohibition of non-canonical Wnt signaling pathway in these cells confirmed by reduced levels of cytosolic co-receptors of FZD, such as LRP5, LRP6, RYK and ROR2, and downstream factors of non-canonical Wnt signaling pathways, such as RhoA, Rac, JNK, ATF2 and p-PKC. These results indicate that FZD8 plays an important role in aberrant activation of the canonical and non-canonical Wnt pathway, and is critical for the proliferation and survival of renal cancer cells.

To further investigate the function of FZD8 in the proliferation and metastasis of RCC, we upregulated the levels of FZD8 in two RCC cell lines. Nevertheless, we did not detect that FZD8 overexpression could promote or inhibit the proliferation and metastasis in RCC cells. In most cases, if the knockdown of some functional protein will prohibit the proliferation or metastasis of some tumor, the overexpression of this protein will promote proliferation and metastasis. Nevertheless, the results of our experiments is different from our common sense, and the underlying mechanism remains unclear.

In summary, we propose that FZD8 activates the canonical and non-canonical Wnt signaling pathway, plays an important role in the development of RCC, making FZD8 a potential RCC diagnosis biomarker and a putative promising drug target for human RCC therapy.

## MATERIALS AND METHODS

### Patients and clinical samples

The tumor and adjacent normal tissues were collected within one hour after surgical resection and immediately stored in liquid nitrogen for further use. All specimens were obtained under guidelines approved by the Ethical Committee of the Second Military Medical University (Shanghai, China). Written informed consent was obtained from every patient at the time of enrollment. None of the patients had undergone preoperative radiotherapy or chemotherapy. Histopathological diagnosis was carried out by two pathologists.

### Immunohistochemistry (IHC)

Immunohistochemistry gets the technical support from the Biolink Biotechnology Co. Ltd. Briefly, tissue sections (4 μm thick) from paraffin-embedded tumor tissues of renal cancer patients were obtained. These sections were deparaffinized, rehydrated, and prepared for antigen retrieval. They were blocked with 10% goat serum and then incubated with primary antibody against FZD8 (1:200, CST) at 4°C; overnight. Second day, the samples were incubated with secondary antibody for 1h at room temperature, and followed with adding DAB and counterstained with hematoxylin (Beyorime Institute of Biotechnology, Inc.).

### Cell lines and reagents

The RCC cell lines A498, ACHN, 786O, 769P, CAKI-1 and CAKI-2 and normal kidney epithelial cell lines 293T and HK2 were purchased from American Type Culture Collection (ATCC, Rockville, MD, USA). 786-O and 769P cells were cultured in RPMI-1640 (Gibco, Thermo Fisher Scientific, Waltham, MA, USA), A498 and ACHN were cultured in MEM (Gibco, USA), CAKI-1 and CAKI-2 cells were cultured in McCoy’s 5A (Gibco, USA), 293T cells were cultured in DMEM (Gibco, USA) supplemented with 10% fetal bovine serum (Gibco, USA), 100 U/ml penicillin, and 100 mg/ml streptomycin (Gibco, USA). HK2 cells were cultured in Keratinocyte Serum Free Medium (K-SFM) supplied with each of the two additives required to grow this cell line, such as bovine pituitary extract (BPE) and human recombinant epidermal growth factor (EGF). Cells were cultured in a humidified incubator in an atmosphere of 95% air and 5% CO_2_ at 37°C.

### RNA extraction and quantitative real-time-PCR (qRT-PCR)

The culture medium was removed, cells were washed with PBS, and total RNA was extracted from renal cancer cell lines and tissues using TRIzol reagent (TaKaRa, Japan) following the manufacturer instructions. RNA concentration was measured at 260 nm in a UV/VIS spectrophotometer from Thermo. Extracted RNA was stored at -80°C. Gene expression was determined using SYBR^®^Green PCR Kit (Takara, Japan) based on Applied Biosystems StepOne-Plus qRT-PCR System. All mRNAs were normalized to the housekeeping gene β-actin, which was amplified as the internal control. Relative quantification expression was calculated according to the comparative method of 2^-ΔΔCT^. Primers were purchased from Sangon Corp. Primer sequences used for human FZD8 were:5’-GGACTACAACCGCACCGACCT-3’(forward)and 5’-ACCACAGGCCGATCCAGAAGAC-3’(reverse). PCR condition: 95°C for 10min, 95°C for 15S and 60°C for 1min for 40 cycles, followed by a final extension at 95°C for 15S, 60°C for 1 min and 95°C for 15S. Samples were tested in triplicate runs.

### Transfection and RNA interference

Human full-length cDNA of FZD8 was cloned into expression plasmid pCMV3-FZD8-Flag (Biolink Biological Inc.). Two FZD8 targeted lentivirus-based shRNA was designed by Biolink Biotechnology Co. Ltd (Shanghai, China) to silence FZD8 mRNA expression in all experiments (the targeted human FZD8 sequences are: (FZD8 shRNA-1) 5’-CTGTGCATGGACTACAACCGC-3’ and (FZD8 shRNA-2) 5’-AAGACAGGCCAGATCGCTAAC-3’ respectively), and control (non-silencing) shRNA (in Puromycine-C-RS vector). Following annealing, double strands of shRNA were inserted into lentiviral pHBLV-U6-Puro vector (Biolink). For lentiviral packaging, HEK-293T cells were co-transfected with the lentiviral vector, and packaging vectors psPAX2 and PMD2G using lipoFiter™ Liposomal Transfection Reagent (Biolink Shanghai, China), according to the manufacturer instructions. 786O and A498 cells were plated in six-well plates with fresh media without antibiotics. When the cells grown to 30-50% confluence, cells were seeded in serum-free media and lentivirus particles were added to the culture media at a multiplicity of infection (MOI) of 30. Cells were grown at 37°C, and 24 hours following transfection cells were placed with fresh media. Transfected cells were re-plated in 6cm dishes for selection with puromycin (10 μg/ml). FZD8 expression in stable transfectants was assayed by qRT-PCR. Stable transfectants were maintained in regular medium for further analysis.

### CCK8 assay

Cell viability was determined by Cell Counting Kit 8 (CCK8, Dojindo, Japan). RCC cells were seeded into 96-well plates at 2000 cells per well. The cells were incubated for 24h, 48h, 72h, 96h and 120h respectively. 10μl CCK-8 reagent (DOJINDO) was added into each well 3h before the end of incubation. Optical density value (OD) of each well was measured to assess the number of viable cells at a wavelength of 450nm. All experiments were performed in quintuplicate and independently repeated 3 times.

### Transwell migration and invasion assays

Transwell migration and invasion assays were carried out using 24-well transwell migration chambers (Corning Life Sciences, NY, USA) with 8-μm pore size polyethylene membranes. For the migration assay, cells were placed in the upper chamber of each insert without Martigel coating. For the invasion assay, cells were placed in the upper chamber of each insert, which was precoated with Matrigel (BD Biosciences, USA). For both assays, RCC cells were starved overnight and trypsinized and suspended in serum-free medium. The 1×10^4^ cells (for the migration assay and invasion assay) were seeded in starvation medium on the top chamber. The bottom chamber was filled with 10%FBS in medium which acted as chemoattractant. After 24h incubation, the cells migrating or invading to the lower chamber were fixed with anhydrous ethanol for 30min, stained with crystal violet solution for 30min, and counted from 10 randomly chosen fields using a microscope. Three independent experiments were performed, and the data are presented as the average±SD.

### Wound-healing assay

786O and A498 cells were plated at 5×10^5^ cells per well in 6-well plates and cultured until the plates reached confluence. Scratches were created in the cell monolayer with a 100μl pipette tip to scrape in a straight streak, washed with PBS and the medium was replaced with serum free medium. Images were captured at 0, 9, and 18h following the initial streak to estimate cell migration.

### Plate colony formation assay

Two hundred individual cells of the 786O and A498 cells were seeded into 6cm plates and cultured in the 37°C; incubator for 10 days until most single colony were composed of more than 100 cells. The plates were washed by PBS, fixed with 10% formalin, and stained with 0.5% crystal violet and counted.

### *In vivo* xenograft tumor formation

The mice experiments were conducted in the animal facility of Second Military University and approved by the Institutional Animal Care and Use committee. Renal cancer xenografts were established with 4-week-old female BALB/c nude mice. Briefly, 786O cells stably transfected with either control or FZD8 shRNA-1 were trypsinized and resuspended in PBS (pH 7.4). The cell suspensions were then mixed with matrigel (Corning) (volume ratio:7:3). The mixture containing 5×10^6^ cells in a volume of 100ul was injected subcutaneously into the flanks of female nude mice (5 mice/group). Tumor volumes in mm^3^ were determined by the formula multiplying by 0.5×width^2^×length.

### Western blot analysis

Cells were lysed with RIPA buffer (beyotime institute of biotechnology; Shanghai, China) with protease inhibitor (Roche, Indianapolis, IN, USA) and total proteins were extracted at 4°C;. Proteins were separated by sodium dodecyl sulfate (SDS)-polyacrylamidegel electrophoresis (PAGE) and then electro-transferred to a polyvinlidene difluoride (PVDF) membrane (Millipore, Bedford, MA, USA). When the protein was transferred onto the membrane, the blot was blocked with 5% non-fat milk in Tris-buffered saline and probed overnight at 4°C; using the following primary antibodies: anti-FZD8 rabbit polyclonal antibody (pAb) (Sigma, Cat No. SAB2100868 and HPA045025, 73kDa), and anti-RYK rabbit monoclonal antibody (mAb) (Cat No. ab124961, 68kDa) were purchased from Abcam Inc (Cambridge, MA, USA). Anti-β-catenin rabbit mAb (Cat No. #8480S, 92kDa), anti-Cyclin D1 rabbit mAb (Cat No. #2978S, 36kDa), anti-C-myc rabbit mAb (Cat No. #13987S, 57-65kDa), anti-Snail rabbit mAb (Cat No. #3879, 29kDa), anti-Vimentin rabbit mAb (Cat No. #5741S, 57kDa), anti-E-Cadherin rabbit mAb (Cat No. #3195S, 135kDa), anti-LRP5 rabbit mAb (Cat No. #5731S, 200kDa), anti-LRP6 rabbit mAb (Cat No. #2560S, 180kDa), anti-ROR2 rabbit mAb (Cat No. #88639S, 135kDa), anti-Rho A rabbit mAb (Cat No. #2117S, 21kDa), anti-Rac 1/2/3 rabbit pAb (Cat No. #2465S, 21kDa), anti-JNK rabbit pAb (Cat No. #9252S, 46 and 54kDa), anti-ATF-2 rabbit mAb (Cat No. #9226S, 70kDa), anti-phospho-PKC α/β II pAb (Cat No. #9375S, 80kDa) and anti-β-actin rabbit mAb (Cat No. #4970S, 45kDa) were purchased from Cell Signaling Technology Inc (Beverly, MA, USA). Appropriate antibodies were used for secondary antibody reaction. Signal was detected by the Western Blot Detcting System (Amersham Imager 600).

### Statistical analysis

Results were presented as the mean±SD for at least three experiments for each group. Statistical software SPSS 19.0 was used for all analysis. All qRT-PCR reactions were run in triplicate, and the mean threshold cycles were figured up. The average expression levels of FZD8 were normalized using β-actin as a reference gene, and 2^-Δct^ method was subsequently applied. The 2^-ΔΔct^ method was used to express the level of FZD8 in RCC tissues and adjacent normal tissues. Two-tailed non-parametric Wilcoxon test was used to estimate the significant difference of FZD8 levels between tumor tissues and adjacent normal tissues. The data derived from cell lines experiments are presented as the mean±SD and evaluated with a two-tailed Student’s t-test. Differences between variables were evaluated using the chi-square test or Fisher’s exact test. Statistical differences were determined using ANOVA and the Student’s t-test for independent samples. Treatment differences were assessed using a Fisher’s Least Significant Difference test [LSD(L)]. *P<0.05 was considered statistically significant, and extremely significant difference **P<0.01 and ***P<0.001.

## SUPPLEMENTARY MATERIALS TABLES



## Abbreviations

RCC: Renal Cell Carcinoma
FZD8: Frizzled 8
EMT: Epithelial to Mesenchymal Transition
APC: Adenomatous Polyposis Coli
VHL: von Hippel-lindau
ccRCC: clear cell Renal Cell Carcinoma
qRT-PCR: quantitive Reverse Transcription-Polymerase Chain Reaction
shRNAs: short hairpin RNAs

## References

[1] Murai M, Oya M (2004). Renal cell carcinoma: etiology, incidence and epidemiology. Current Opinion in Urology.

[2] Berktaş B, Siegel R, Ma J, Zou Z, Jemal A (2014). Cancer statistics, 2014. CA Cancer J Clin.

[3] Thievessen I, Schulz WA, Seifert HH, Swiatkowski S, Florl AR (2003). E-cadherin involved in inactivation of WNT/beta-catenin signalling in urothelial carcinoma and normal urothelial cells. British Journal of Cancer.

[4] Bates RC, Mercurio A (2005). The epithelial-mesenchymal tansition (EMT) and colorectal cancer progression. Cancer Biology & Therapy.

[5] Berx G, Van RF (2001). The E-cadherin/catenin complex: an important gatekeeper in breast cancer tumorigenesis and malignant progression. Breast Cancer Research.

[6] Wakatsuki SJ, Watanabe R, Saito K, Saito T, Katagiri A, Sato S, Tomita Y (1996). Loss of human E-cadherin (ECD) correlated with invasiveness of transitional cell cancer in the renal pelvis, ureter and urinary bladder. Cancer Letters.

[7] Huber MA, Kraut N, Beug H (2005). Molecular requirements for epithelial-mesenchymal transition during tumor progression. Curr Opin Cell Biol.

[8] Guarino M, Rubino B, Ballabio G (2007). The role of epithelial-mesenchymal transition in cancer pathology. Pathology.

[9] Vider BZ, Zimber A, Chastre E, Prevot S, Gespach C, Estlein D, Wolloch Y, Tronick SR, Gazit A, Yaniv A (1996). Evidence for the involvement of the Wnt 2 gene in human colorectal cancer. Oncogene.

[10] Peruzzi B, Athauda G, Bottaro DP (2006). The von Hippel–Lindau tumor suppressor gene product represses oncogenic β-catenin signaling in renal carcinoma cells. Proceedings of the National Academy of Sciences of the United States of America.

[11] Anastas JN, Moon RT (2013). WNT signalling pathways as therapeutic targets in cancer. Nature Reviews Cancer.

[12] Holland JD, Klaus A, Garratt AN, Birchmeier W (2010). Wnt signaling in stem and cancer stem cells. Seminars in Cell & Developmental Biology.

[13] Cai J, Guan H, Fang L, Yang Y, Zhu X, Yuan J, Wu J, Li M (2013). MicroRNA-374a activates Wnt/β-catenin signaling to promote breast cancer metastasis. Journal of Clinical Investigation.

[14] Hirata H, Ueno K, Nakajima K, Tabatabai ZL, Hinoda Y, Ishii N, Dahiya R (2013). Genistein downregulates onco-miR-1260b and inhibits Wnt-signalling in renal cancer cells. British Journal of Cancer.

[15] Ueno K, Hirata H, Hinoda Y, Dahiya R (2013). Frizzled homolog proteins, microRNAs and Wnt signaling in cancer. International journal of cancer.

[16] Pode-Shakked N, Metsuyanim S, Rom-Gross E, Mor Y, Fridman E, Goldstein I, Amariglio N, Rechavi G, Keshet G, Dekel B (2009). Developmental tumourigenesis: NCAM as a putative marker for the malignant renal stem/progenitor cell population. Journal of Cellular & Molecular Medicine.

[17] Rhee CS, Sen M, Lu D, Wu C, Leoni L, Rubin J, Corr M, Carson DA (2002). Wnt and frizzled receptors as potential targets for immunotherapy in head and neck squamous cell carcinomas. Oncogene.

[18] Bazhin A, Tambor V, Dikov B, Philippov P, Schadendorf D, Eichmuller S (2010). cGMP-phosphodiesterase 6, transducin and Wnt5a/Frizzled-2-signaling control cGMP and Ca(2+) homeostasis in melanoma cells. Cellular & Molecular Life Sciences Cmls.

[19] Lee EH, Chari R, Lam A, Ng RT, Yee J, English J, Evans KG, Macaulay C, Lam S, Lam WL (2008). Disruption of the non-canonical WNT pathway in lung squamous cell carcinoma. Clinical Medicine Oncology.

[20] Bian Y, Chang X, Liao Y, Wang J, Li Y, Wang K, Wan X (2016). Promotion of epithelial-mesenchymal transition by Frizzled2 is involved in the metastasis of endometrial cancer. Oncology Reports.

[21] Zins K, Schäfer R, Paulus P, Dobler S, Fakhari N, Sioud M, Aharinejad S, Abraham D (2016). Frizzled2 signaling regulates growth of high-risk neuroblastomas by interfering with β-catenin-dependent and β-catenin-independent signaling pathways. Oncotarget.

[22] Janssens N, Andries L, Janicot M, Perera T, Bakker A (2004). Alteration of frizzled expression in renal cell carcinoma. Tumor Biology.

[23] Thiele S, Rauner M, Goettsch C, Rachner TD, Benad P, Fuessel S, Erdmann K, Hamann C, Baretton GB, Wirth MP (2011). Expression profile of WNT molecules in prostate cancer and its regulation by aminobisphosphonates. Journal of Cellular Biochemistry.

[24] Caldwell GM, Jones CE, Soon Y, Warrack R, Morton DG, Matthews GM (2008). Reorganisation of Wnt-response pathways in colorectal tumorigenesis. British Journal of Cancer.

[25] Yan J, Liu T, Zhou X, Dang Y, Yin C, Zhang G (2016). FZD6, targeted by miR-21, represses gastric cancer cell proliferation and migration via activating non-canonical wnt pathway. Am J Transl Res.

[26] García-Castro B, Alvarez-Zavala M, Riveros-Magaña AR, Ortíz-Lazareno PC, Ratkovich-González S, Hernández-Flores G, Bravo-Cuellar A, Jave-Suarez LF, Aguilar-Lemarroy A (2013). Restoration of WNT4 inhibits cell growth in leukemia-derived cell lines. Bmc Cancer.

[27] Asad M, Wong MK, Tan TZ, Choolani M, Low J, Mori S, Virshup D, Thiery JP, Huang RY (2014). FZD7 drives *in vitro* aggressiveness in Stem-A subtype of ovarian cancer via regulation of non-canonical Wnt/PCP pathway. Cell Death & Disease.

[28] Anastas JN, Kulikauskas RM, Tamir T, Rizos H, Long GV, Euw EMV, Yang PT, Chen HW, Haydu L, Toroni RA (2014). WNT5A enhances resistance of melanoma cells to targeted BRAF inhibitors. Journal of Clinical Investigation.

[29] Deng B, Zhang S, Yuan M, Zhang Y, Fang W, Guo K (2015). Down-regulation of Frizzled-7 expression inhibits migration, invasion, and epithelial–mesenchymal transition of cervical cancer cell lines. Medical Oncology.

[30] Xu R, Zeng S, Xie W, Sun C, Chen YL, Chen MJ, Zhang L (2016). The expression and function of Frizzled-7 in human renal cell carcinoma. Clinical & Translational Oncology.

[31] Zhang Z, Schittenhelm J, Guo K, Bühring HJ, Trautmann K, Meyermann R, Schluesener HJ (2006). Upregulation of frizzled 9 in astrocytomas. Neuropathology & Applied Neurobiology.

[32] Sun S, Liu S, Duan SZ, Zhang L, Zhou H, Hu Y, Zhou X, Shi C, Zhou R, Zhang Z (2014). Targeting the c-Met/FZD8 signaling axis eliminates patient-derived cancer stem-like cells in head and neck squamous carcinomas. Cancer Research.

[33] Yin S, Xu L, Bonfil RD, Banerjee S, Sarkar FH, Sethi S, Reddy KB (2013). Tumor Initiating Cells and FZD8 play a major role in drug resistance in Triple-Negative Breast Cancer. Molecular Cancer Therapeutics.

[34] Demirci U, Boyacıoğlu SÖ, Kasap E, Bilgiç F, Gerçeker E, Yıldırım H, Baykan A, Ellidokuz E, Yüceyar M, Korkmaz M (2016). P-014Are RHOA, CSNK1A1, DVL2, FZD8 and LRP5 Genes Novel Biomarkers in the conversion from Intestinal Metaplasia to Gastric Cancer?. Annals of Oncology.

[35] Jiang Q, He M, Guan S, Ma M, Wu H, Yu Z, Jiang L, Wang Y, Zong X, Jin F (2016). MicroRNA-100 suppresses the migration and invasion of breast cancer cells by targeting FZD-8 and inhibiting Wnt/β-catenin signaling pathway. Tumor Biology.

[36] Fu X, Li H, Liu C, Hu B, Li T, Wang Y (2016). Long noncoding RNA AK126698 inhibits proliferation and migration of non-small cell lung cancer cells by targeting Frizzled-8 and suppressing Wnt/β-catenin signaling pathway. Onco Targets Ther.

[37] Li ZG, Yang J, Vazquez ES, Rose D, Vakar-Lopez F, Mathew P, Lopez A, Logothetis CJ, Lin SH, Navone NM (2007). Low-density lipoprotein receptor-related protein 5 (LRP5) mediates the prostate cancer-induced formation of new bone. Oncogene.

[38] Li Y, Lu W, He X, Schwartz AL, Bu G (2004). LRP6 expression promotes cancer cell proliferation and tumorigenesis by altering beta-catenin subcellular distribution. Oncogene.

[39] Katso RM, Manek S, Ganjavi H, Biddolph S, Charnock MF, Bradburn M, Wells M, Ganesan TS (2000). Overexpression of H-Ryk in epithelial ovarian cancer: prognostic significance of receptor expression. Clinical Cancer Research.

[40] Henry C, Llamosas E, Knipprath-Mészáros A, Schoetzau A, Obermann E, Fuenfschilling M, Caduff R, Fink D, Hacker N, Ward R (2015). Targeting the ROR1 and ROR2 receptors in epithelial ovarian cancer inhibits cell migration and invasion. Oncotarget.

[41] Gordon MD, Nusse R (2006). Wnt signaling: multiple pathways, multiple receptors, and multiple transcription factors. Journal of Biological Chemistry.

[42] Veeman MT, Axelrod JD, Moon RT (2003). A second canon. Functions and mechanisms of beta-catenin-independent Wnt signaling. Dev Cell.

[43] Prgomet Z, Axelsson L, Lindberg P, Andersson T (2015). Migration and invasion of oral squamous carcinoma cells is promoted by WNT5A, a regulator of cancer progression. Journal of Oral Pathology & Medicine.

[44] Banumathy G, Cairns P (2010). Signaling pathways in renal cell carcinoma. Cancer Biology & Therapy.

[45] Peifer M, Polakis P (2000). Wnt signaling in oncogenesis and embryogenesis—a look outside the nucleus. Science.

[46] King TD, Zhang W, Suto MJ, Li Y (2012). Frizzled7 as an emerging target for cancer therapy. Cellular Signalling.

[47] Saini S, Majid S, Dahiya R (2011). The complex roles of Wnt antagonists in RCC. Nature Reviews Urology.

[48] Ma J, Lu W, Chen D, Bo X, Li Y (2017). Role of Wnt Co ^–^ receptor LRP6 in Triple Negative Breast Cancer Cell Migration and Invasion. Journal of Cellular Biochemistry.

[49] Ren D, Chen J, Zhi L, Yan H, Yan Y, Da W, Zhang J, Ao L, Bo C, Ito TK (2015). LRP5/6 directly bind to Frizzled and prevent Frizzled-regulated tumour metastasis. Nature Communications.

[50] Adamo A, Fiore D, De Martino F, Roscigno G, Affinito A, Donnarumma E, Puoti I, Ricci-Vitiani L, Pallini R, Quintavalle C, Condorelli G (2017). RYK promotes the stemness of glioblastoma cells via the WNT/ β-catenin pathway. Oncotarget.

[51] Lu C, Wang X, Zhu H, Jian F, Ni S, Huang J (2015). Over-expression of ROR2 and Wnt5a cooperatively correlates with unfavorable prognosis in patients with non-small cell lung cancer. Oncotarget.

[52] Sun B, Ye X, Lin L, Shen M, Jiang T (2015). Up-regulation of ROR2 is associated with unfavorable prognosis and tumor progression in cervical cancer. International Journal of Clinical & Experimental Pathology.

[53] Henry CE, Llamosas E, Djordjevic A, Hacker NF, Ford CE (2016). Migration and invasion is inhibited by silencing ROR1 and ROR2 in chemoresistant ovarian cancer. Oncogenesis.

[54] Merle P, Monte SDL, Kim M, Herrmann M, Tanaka S, Bussche AVD, Kew MC, Trepo C, Wands JR (2004). Functional consequences of frizzled-7 receptor overexpression in human hepatocellular carcinoma. Gastroenterology.

[55] Qi J, Yu Y, Akilli ÖÖ, Holland JD, Besser D, Fritzmann J, Wulf-Goldenberg A, Eckert K, Fichtner I, Birchmeier W (2015). New Wnt/β-catenin target genes promote experimental metastasis and migration of colorectal cancer cells through different signals. Gut.

[56] Sherwood V (2015). WNT signaling: an emerging mediator of cancer cell metabolism?. Molecular & Cellular Biology.

[57] Liu D, Mei X, Wang L, Yang X (2017). RhoA inhibits apoptosis and increases proliferation of cultured SPCA1 lung cancer cells. Molecular Medicine Reports.

[58] Chang HR, Nam S, Lee J, Kim JH, Jung HR, Park HS, Park S, Ahn YZ, Huh I, Balch C (2016). Systematic approach identifies RHOA as a potential biomarker therapeutic target for Asian gastric cancer. Oncotarget.

[59] Katz E, Sims AH, Sproul D, Caldwell H, Dixon MJ, Meehan RR, Harrison DJ (2012). Targeting of Rac GTPases blocks the spread of intact human breast cancer. Oncotarget.

[60] Tournier C (2013). The 2 Faces of JNK Signaling in Cancer. Genes Cancer.

[61] Wu D, Chen C, Wu Z, Liu B, Gao L, Yang Q, Chen W, Chen J, Bao Y, Qu L (2016). ATF2 predicts poor prognosis and promotes malignant phenotypes in renal cell carcinoma. Journal of Experimental & Clinical Cancer Research.

[62] Wang HQ, Xu ML, Ma J, Zhang Y, Xie CH (2011). Frizzled-8 as a putative therapeutic target in human lung cancer. Biochemical & Biophysical Research Communications.

[63] Khan NI, Bradstock KF, Bendall LJ (2007). Activation of Wnt/beta-catenin pathway mediates growth and survival in B-cell progenitor acute lymphoblastic leukaemia. British Journal of Haematology.

