# Supplementary material for: Frizzled 8 promotes the cell proliferation and metastasis of renal cell carcinoma
Source: Oncotarget. 2017 Sep 8;8(45):78989–9002. doi: 10.18632/oncotarget.20742 (PMC5668014; doi:10.18632/oncotarget.20742)
Supplement: Supplementary file 1 [file oncotarget-08-78989-s001.pdf]

Frizzled 8 promotes the cell proliferation and metastasis of renal cell carcinoma

SUPPLEMENTARY MATERIALS

Supplementary Table 1: Characteristics of RCC tissues and peritumoral tissues from the 20 patients

		Cases (n=20)
Age (y) (mean±SD)		61.8±10
Gender	Males	14 (70%)
	Females	6 (30%)
Grade		
	1	5 (25%)
	2	12 (60%)
	3+4	3 (15%)
pT		
	1	9 (45%)
	2	4 (20%)
	3	5 (25%)
	4	2 (10%)
pN		
	(-)	18 (90%)
	(+)	2 (10%)
pM		
	(-)	18 (90%)
	(+)	2 (10%)
Pathology		
	Clear cell carcinoma	20 (100%)

Abbreviations: pT, pathological tumor classification; pN, lymph node invasion; pM, distant metastasis.

Supplementary Table 2: The data from the nude mice experiments

	control shRNA	FZD8 shRNA	P
**tumor volume (mm^3)**			
week 1	100±35	100±22	0.99
week 2	168±44	130±43	0.2
week 3	225±51	150±56	0.06
week 4	356±62	178±74	0.028
week 5	587±78	220±85	0.007
week 6	698±112	299±123	0.003
tumor weight (mg)	191±29.48	102.67±24.86	0.0002
body weight (g)			
week 0	20.35±0.47	20.1±0.81	0.53
week 1	21.23±0.52	21±0.58	0.48
week 2	21.98±0.53	21.83±0.52	0.63
week 3	22.43±0.50	22.6±0.6	0.61
week 4	23.18±0.48	23.3±0.61	0.72
week 5	24.02±0.46	24.13±0.55	0.69
week 6	24.65±0.34	25.08±0.50	0.11
